# The complete mitogenome and phylogeny analysis of *Pseudohemiculter hainanensis* (Boulenger, 1900) (Cyprinidae: Cultrinae)

**DOI:** 10.1080/23802359.2022.2151828

**Published:** 2022-12-09

**Authors:** Yifei Wang, Jiayang He, Zhiqiang Wu, Liangliang Huang, Minghui Gao, Mingsi Li, Jie Feng

**Affiliations:** a College of Environmental Science and Engineering, Guilin University of Technology, Guilin, China; b Collaborative Innovation Center for Water Pollution Control and Water Safety in Karst Area, Guilin University of Technology, Guilin, China; c Guangxi Key Laboratory of Environmental Pollution Control Theory and Technology, Guilin, China; d Ministry of Water Resources, Bureau of Hydrology and Water Resources of Pearl River Water Conservancy Commission, Guangzhou, China

**Keywords:** *Pseudohemiculter hainanensis*, mitogenome, phylogeny

## Abstract

The *Pseudohemiculter hainanensis* (Boulenger, 1900) is a small Cyprinidae fish that has a wide distribution in China. In this study, we characterized the complete mitochondrial genome of *P. hainanensis* by the Illumina NovaSeq sequencing platform in Guangxi, China. The assembled mitogenome is 16,647 base pairs (bp) and consists of 13 protein-coding genes (PCGs), 22 transfer RNAs, two ribosomal RNAs, and a control region (D-loop). Nucleotide composition of the complete mitogenome is 29.69% (A), 24.82% (T), 27.97% (C), and 17.52% (G), with an A + T bias of 54.51%. The maximum-likelihood tree based on 13 PCGs showed that *Pseudohemiculter hainanensis* formed an independent lineage and *P. hainanensis* was closer to *T. houdemeri.*

## Introduction

The *Pseudohemiculter hainanensis* (Boulenger, 1900) is a small Cyprinidae fish, that is widely distributed in the drainage basins of the Yuanjiang River, the Pearl River, Hainan Island, and the middle reaches of the Yangtze River in China. It is a  freshwater fish that lives in the upper middle layer of the water (Luo and Chen 1998). *P. hainanensis* was classified as least concern (LC) according to International Union for Conservation of Natures. No research on *P. hainanensis* has been documented till date; therefore, it is highly important to obtain the complete mitochondrial genome of *P. hainanensis* for further studies. This study obtained the complete mitochondrial genome of *P. hainanensis*, analyzed the structural features of the complete mitochondrial genome, and explored the phylogenetic relationships within Cultrinae to provide a basis for further studies on the genetic evolution and classification of *P. hainanensis*.

## Materials

Specimen of *P. hainanensis* was collected from the farmers market of Laibin, Guangxi Zhuang Autonomous Region, China (23°43′24″N, 109°13′45″E) on 5 July 2021. The species was carefully distinguished as ‘Cypriniformes, Cyprinidae, Cultrinae, and *Pseudohemiculter*’ according to *Culterinae. Fauna Sinica*, *Osteichthyes*, *Cypriniformes II* book (Luo and Chen 1998). The voucher samples were deposited at the college of environmental science and engineering, Guilin University of Technology (Zhiqiang Wu, wuzhiqiang@glut.edu.cn, under the voucher number pha202105).

## Methods

One *P. hainanensis* captured in 2021 was dissected using a sterilized dissection tool, and 5 g of its dorsal muscle was taken and stored in a centrifuge tube at −80 °C in the refrigerator. Total genomic DNA was extracted using the cetyltrimethylammonium bromide (CTAB) method (Chapela et al. 2007). The extracted DNA was used to construct the library by the Whole Genome Shotgun (WGS) method. Covaris M220 ultrasonic fragmentation machine was used to split the fragments into 400 bp lengths fragments with splices at both ends, and then bridge PCR amplification was performed. The constructed library was sequenced paired-end (PE) using next-generation sequencing (NGS) based on the Illumina NovaSeq sequencing platform. A5-miseq v20150522 (Coil et al. 2015) and spadesv3.9.0 were used to *de novo* assemble high-quality second-generation sequencing data to construct contig and scaffold sequences. The spliced genome sequence was uploaded to the MITOS web (http://mitos.bioinf.uni-leipzig.de/) server for functional annotation (Bernt et al. 2013). Among them, the genetic code is set to 02 vertebrate, and the rest are set according to the default parameters set by MITOS.

## Results

**Figure 1.**
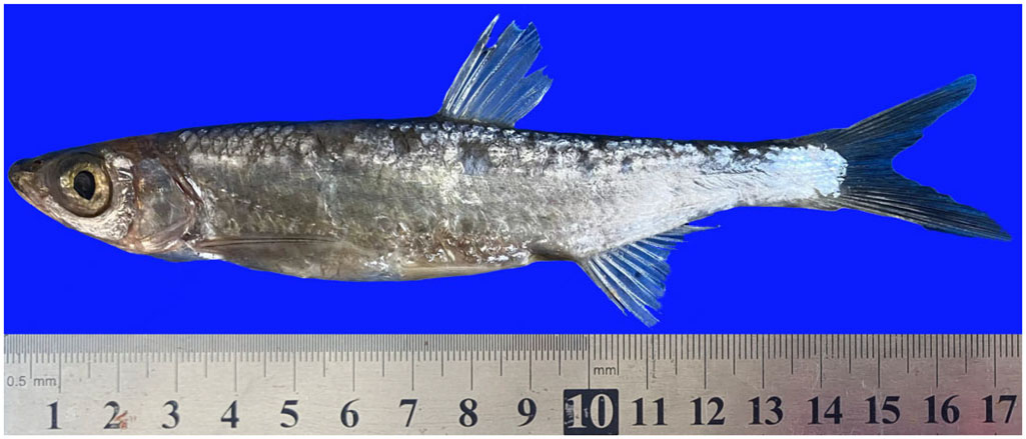
The photo of *P. hainanensis* (this photo was taken by Yifei Wang, the first author of this article).

**Figure 2.**
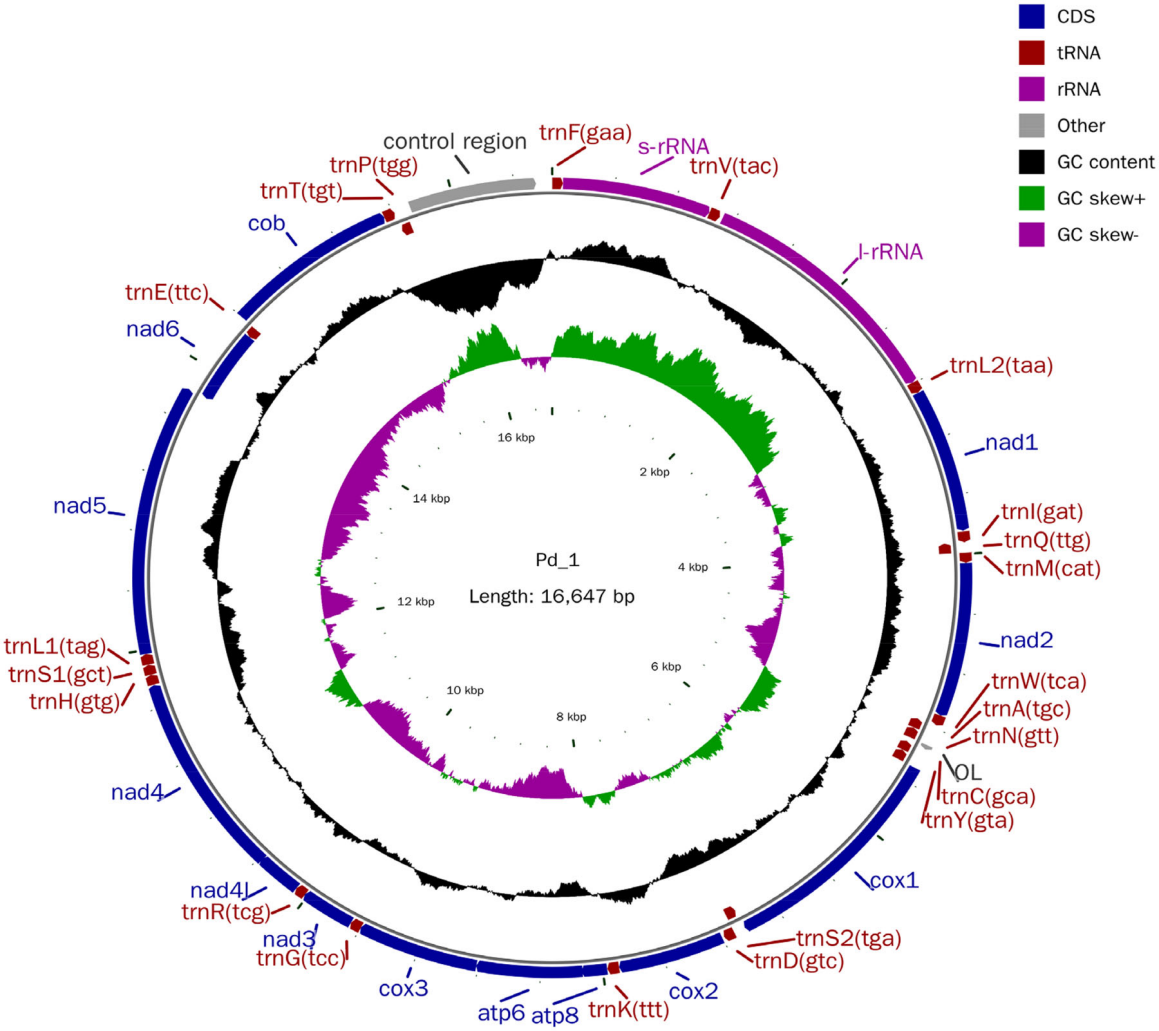
The mitochondrial genome mapping of *P. hainanensis.*

The *P. hainanensis*’s body length is 5–5.6 times its height and *P. hainanensis*’s pectoral fin has 15 branching fins with a scaleless keel between ventrals and anal. The photo of the sample was taken by us in the field and is shown in Figure 1. The complete mitogenome of *P. hainanensis* was sequenced to be 16,647 bp in length and exhibits an average GC content of 45.49%. The complete mitochondrial genome comprised 13 protein-coding genes (PCGs), 22 transfer RNA genes, two ribosomal RNA, and a control region (D-loop). Most PCGs of *P. hainanensis* were encoded on the N-strand, except for *ND*6 which was encoded on the J-strand. The overall base composition was 29.69% (A), 24.82% (T), 27.97% (C), and 17.52% (G), with an A + T bias of 54.51%. Twelve genes started with ATG while only *CO*I started with GTG among the 13 PCGs. Eight genes shared the termination codon TAA including *ND*2, *CO*I, *CO*II, *CO*III, *ATP*6, *ND*4, *ND*4*L*, and *Cytb*, and the gene with TAG as the stop codon was *ND*1, *ATP*8, *ND*3, *ND*5, and *ND*6. The genome map is shown in Figure 2.

**Figure 3.**
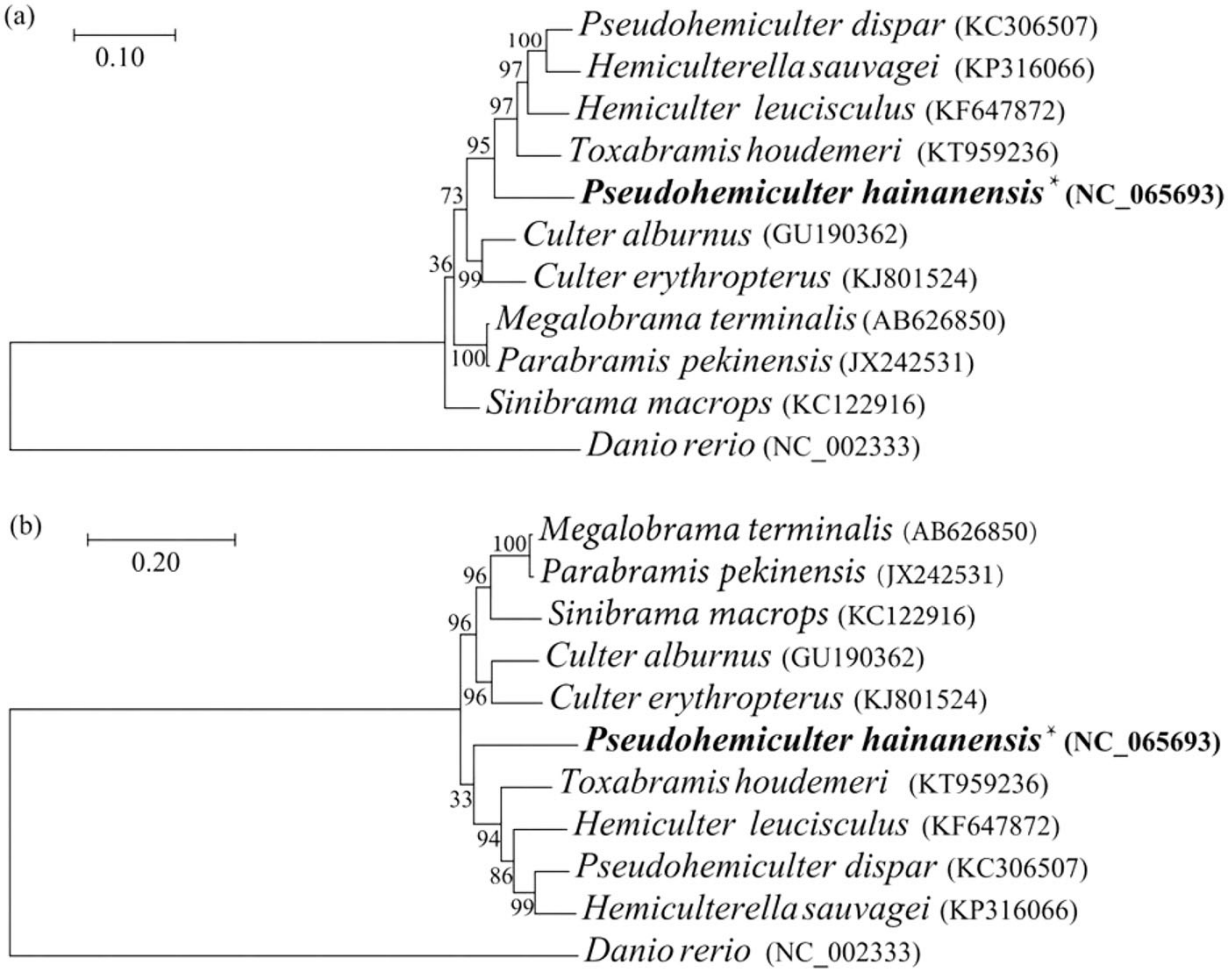
Maximum-likelihood trees showing the phylogenetic relationships among Cultrinae species based on 13 protein-coding genes (a) and amino acids (b).

To investigate the phylogenetic relationships of *P. hainanensis* and other 10 species from Cultrinae, phylogenetic trees were obtained using maximum-likelihood (ML) analyses based on the combined DNA sequences of 13 PCGs and corresponding amino acids (Ai et al. 2013; Imoto et al. 2013; Xiang et al. 2013; Zhang et al. 2014; Chen, Wang, et al. 2016; Chen, Li, et al. 2016; He et al. 2016; Wang et al. 2017). *Danio rerio* from Danioninae was selected as an outgroup. ML trees were based on 13 PCGs (Figure 3(a)), while ML trees were on the basis of amino acids (Figure 3(b)). The phylogenetic analysis showed that all fishes of the genus Cultrinae were clustered into one group. Both trees showed that *P. hainanensis* formed an independent lineage and *P. hainanensis* was closer to *T. houdemeri.*

## Discussion and conclusions

The gene arrangement pattern and translated orientation of *P. hainanensis* were identical to most vertebrates (Boore 1999). The phylogenetic analysis showed that *P. hainanensis* and *Toxabramis houdemeri* were in the same proximal branch, and *P. hainanensis* was differentiated earlier. Dai et al. (2005) used the characteristics of bone differences to study the phylogeny of Cultrinae. They found that genetic differentiation between *Pseudohemiculter*, *Toxabramis*, and *Hemiculter* was small and the genetic relationship was close. All of the above showed that *P. hainanensis and T. houdemeri* may have similar genetic backgrounds and need further research.

The results of this study showed that *P. hainanensis* formed an independent lineage and *P. hainanensis* was closer to *T. houdemeri*. This study can provide important basic information for species identification, geographic population division, and kinship identification of *P. hainanensis* in the freshwater waters of China.

## Data Availability

The genome sequence data that support the findings of this study are openly available in GenBank of NCBI at https://www.ncbi.nlm.nih.gov/ under the accession number NC_065693. The associated BioProject, SRA, and Bio-Sample numbers of specimen are PRJNA832045, SRR18919243, and SAMN27773197, respectively.
